# The Acceptance of COVID-19 Vaccination Under Different Methods of Investigation: Based on Online and On-Site Surveys in China

**DOI:** 10.3389/fpubh.2021.760388

**Published:** 2021-11-25

**Authors:** Yun Lyu, Xiaozhen Lai, Xiaochen Ma, Lei Cao, Hong Lei, Jiahao Wang, Haijun Zhang, Rize Jing, Huangyufei Feng, Jia Guo, Li Li, Hai Fang

**Affiliations:** ^1^School of Public Health, Peking University, Beijing, China; ^2^China Center for Health Development Studies, Peking University, Beijing, China; ^3^National Immunization Program, Chinese Center for Disease Control and Prevention, Beijing, China; ^4^Peking University Health Science Center—Chinese Center for Disease Control and Prevention Joint Center for Vaccine Economics, Beijing, China; ^5^Key Laboratory of Reproductive Health, National Health Commission of the People's Republic of China, Beijing, China

**Keywords:** COVID-19, vaccine acceptance, online survey, on-site survey, China

## Abstract

As Coronavirus Disease-2019 (COVID-19) vaccines became available in December 2020, increasingly more surveys were organized to examine the acceptance of vaccination, while most of them were conducted online. This study aimed to explore the difference between online and traditional on-site surveys in terms of COVID-19 vaccine acceptance. From November to December 2020, an online survey (*n* = 2013) and an on-site survey (*n* = 4,316) were conducted simultaneously in China. Multivariate logistic regression was used to identify influencing factors of acceptance, and propensity score matching (PSM) was adopted to balance the outcomes. As a result, 90.0% of the online respondents accepted COVID-19 vaccination, while it was only 82.1% in the on-site survey. After applying PSM, the acceptance rate of the on-site survey was declined to 78.6%. The age structure, residence location, education, and health status were observed as important factors in addressing vaccination acceptance, which needed to be specifically considered when designing online surveys.

## Introduction

Since firstly identified in December 2019, Coronavirus Disease-2019 (COVID-19) has spread globally with a severe situation (1–3). The pandemic has resulted in 177.1 million confirmed cases and 3.8 million deaths worldwide as of June 18, 2021 (2), which might have profound impacts on healthcare systems and public health management mechanisms (4, 5). With debates intensifying about lockdowns around the world, vaccines were regarded as the most effective weapon to effectively control the public health crisis. The research and development of vaccines against COVID-19 have been accelerating at an unprecedented speed. By November 2020, multiple candidate vaccines had been tested in the final stage (6), such as those developed by Pfizer-BioNTech, Oxford-AstraZeneca, Sinopharm, and Sinovac (7, 8). It was announced by the Chinese government on December 31, 2020 that COVID-19 vaccination would be available free of charge for Chinese citizens. This achievement has provided confidence to the global fight against COVID-19, which has also provided strong support to ease the pandemic in China and return to normal economic development.

The success of the vaccination program is dependent on the willingness of the general public to get vaccinated. A small number of studies have surveyed adults to estimate public acceptance of COVID-19 vaccination online during the early period of the pandemic. For example, the acceptance rates could reach around 90% in China, 85% in Brazil, 80% in South Africa and South Korea, which in Russia (55%) and France (60%) were lower (9–13). The pandemic in most countries was still severe with some difficulties in conducting on-site surveys, as the spread rate and infection rate among the countries and regions worldwide have continuously been on the rise. Based on the weekly report of the European Center for Disease Prevention and Control (ECDC) as on October 14, 2021, there have been 238,460,430 reported cases and a total of 4,855,764 deaths recorded in 219 countries, territories, and international conveyance (14, 15). Under this situation, most countries had implemented strict measures to control the pandemic, such as lockdowns, restriction in the movements and gathering, social distancing, and quarantining (16). Currently, with the “second-wave” of COVID-19 cases, some governments have considered and implemented further lockdowns to control the pandemic, such as the USA, Italy, Spain, France, and Australia (17, 18). Due to the recurrence of the COVID-19 pandemic and the restrictions in social distancing, an online survey becomes the most ideal method to survey and obtain vaccination acceptance. Although hit by COVID-19 at the first, China has effectively brought the pandemic under control (19–23). Due to the alleviation of the pandemic by joint efforts of the Chinese government and citizens, large-scale on-site surveys became attainable in China in the last quarter of 2020.

As explained in a previous review, online surveys can be subject to considerable bias. Bias can be resulted from the non-representative nature of the online participants with self-selection under the inevitable volunteer effect (24). Since two important components of survey methodology, sample selection and question validation, are frequently overlooked in online surveys, results generated online may be neither replicable nor robust (25). Due to the social distancing requirement in most countries, conducting online surveys is the most appropriate method during the pandemic, enabled with flexibility, automation, timeliness, and lower cost (26). However, the lack of face-to-face interviewers could become a disadvantage with an accumulation of non-representative or biased responses, which is exactly what traditional on-site surveys could address.

Therefore, this study investigated the differences in individual acceptance generated from an online and an on-site survey toward the COVID-19 vaccination, which were conducted simultaneously in China. This study is the very first survey conducted in China and in the world at the very beginning of vaccination approval and before roll-out in China and globally. The aim of this study was to compare the online and on-site field survey results toward the public acceptance of COVID-19 vaccination, which is also groundbreaking with the first large-scale field survey in China and in the world to investigate the public acceptance toward COVID-19 vaccination. It should be noted that the acceptance rate addressed in this study might be different from the up-to-date acceptance rate after nearly a year of routine vaccination. By examining the results under the two survey methods, we targeted to alert global researchers of the potential biases in online surveys and emphasize the principles of survey research, which should be applied in online surveys. More practically, the appropriate statistical methodology would be suggested in this study to reduce bias and enhance rigor, which could be adjusted to more accurate and close-to-reality results when conducting an online survey during the pandemic.

## Materials and Methods

### Study Design, Population, and Sampling

From November 12, 2020 to December 15, 2020, we simultaneously conducted an online survey and an on-site survey among adult residents living in Mainland China. The cross-sectional anonymous online survey was done on the largest Chinese online survey platform, Wen Juan Xing (Changsha Ranxing Information Technology Co., Ltd., Hunan, China). The sample database of Wen Juan Xing consists of over 2.6 million respondents with confirmed personal information. This allows us to collect authentic and representative samples. A total of 2,013 respondents were enrolled in the online survey after excluding incomplete and invalid questionnaires.

Meanwhile, we conducted a cross-sectional on-site survey in five provinces/municipalities in China, namely, Guangdong, Zhejiang, Hubei, Jilin, and Chongqing. A random sampling method was adopted in the on-site survey, and a total of 4,316 valid questionnaires were collected after quality control and manual check procedures. Samples in the two cross-sectional surveys were merged for analysis. The two surveys were approved by Peking University Institutional Review Board (IRB00001052-20011).

### Measures

The online and on-site questionnaires were designed according to a previous study on COVID-19 vaccine acceptance (27) and other studies estimated the acceptance of vaccination against emerging infectious diseases (28–31). Information collected in the questionnaires included (1) socio-demographic characteristics of respondents, such as age, gender, and living residence and (2) acceptance and attitude for COVID-19 vaccination. Most questions were closed-ended and treated as categorical variables. In this study, the primary outcome measure was the acceptance of the COVID-19 vaccination. Based on the question “If a COVID-19 vaccine is successfully developed and approved for using in the future, would you accept the vaccination,” respondents were classified into vaccine accepted group or the refused group in the surveys.

### Statistical Analysis

Descriptive statistics were applied to present some baseline characteristics of respondents enrolled in the surveys, in which categorical variables were displayed as frequencies and proportions. The monetary amount used in the questionnaire was Chinese yuan (CNY). We provided an equivalence in the US Dollars (USD) at an exchange rate of 6.52 yuan per dollar in 2021. To identify the influencing factors of vaccine acceptance for different types of surveys, multivariate logistic regressions were conducted between the online and on-site sample, also for the two surveys respectively. The odds ratio (OR) and 95% CI were calculated and reported.

The propensity score matching (PSM) method was used to match “post-randomization” to directly compare the vaccination acceptance in online and on-site surveys. In the present study, the two samples varied in many socio-economic characteristics, therefore, it is difficult to distinguish the impact of survey type from other factors. The PSM proposed by Rosenbaum and Rubin can create a quasi-random process to correct for selection bias (32), providing an alternative for estimating treatment effects when systematic differences between groups are not random (33). The logit model is recommended to estimate the propensity score (34), which was also adopted in the present study by matching 12 covariates, such as age distribution, gender, region, living residence, marital status, education level, employment, household income, household size (the existence of the elderly was separately analyzed), and health status (the prevalence chronic disease was separately analyzed). The common ranges of propensity score and the standardized bias were graphed. A two-sided *p*-value below 0.05 was considered statistically significant in the present study. All data were analyzed using STATA, version 14.0 (Stata Corp, College Station, TX, USA) with two-tailed tests.

## Results

### Respondent Characteristics

Table 1 presents the basic characteristics of respondents. In the online survey, a total of 2,013 respondents completed the online questionnaires. Respondents were located in all 31 provincial administrative regions of Mainland China. The majority of them (98.3%) were under 60 years old, although we had tried to include as many participants aged above 60 years old as we could. Among the respondents, 49.0% were female, 72.3% were married, 85.1% were employed, and 65.1% resided in eastern China. For educational level, 34.6% of respondents had a high school and below degree, and 65.4% had an associate or bachelor degree and above. Total 68.0% of the respondents thought their health status were good or very good, and 87.4% of the respondents did not report any chronic diseases. Around half of the respondents (48.6%) had an annual family income of CNY 50,000–150,000 (USD 7,670–23,010). The respondents mainly (60.9%) lived in a family of 3–4 members and 55.0% of them did not live with the elderly.

**Table 1:** Characteristics of survey respondents and the number of respondents who accepted vaccination.

**Characteristics**	**Online survey respondents**		**On-site survey respondents**	
	**Total**	**Accept vaccination**	**Total**	**Accept vaccination**
	***N* (%)**	***N* (%)**	***N* (%)**	***N* (%)**
Total	2,013 (100.0)	1,812 (90.0)	4,316 (100.0)	3,543 (82.1)
Age group				
18~30	766 (38.1)	686 (34.1)	727 (16.9)	631 (14.6)
31~40	717 (35.6)	659 (32.7)	609 (14.1)	519 (12.0)
41~50	360 (17.9)	323 (16.1)	515 (11.9)	431 (10.0)
51~60	135 (6.7)	113 (5.6)	591 (13.7)	499 (11.6)
>60	35 (1.7)	31 (1.5)	1,874 (43.4)	1,463 (33.9)
Gender				
Female	987 (49.0)	891 (44.3)	1,869 (43.4)	1,526 (35.4)
Male	1,026 (51.0)	921 (45.8)	2,447 (55.6)	2,017 (46.7)
Living residence				
Urban	1,680 (83.5)	1,513 (74.2)	2,773 (64.2)	2,286 (53.0)
Rural	333 (16.5)	299 (14.6)	1,543 (35.8)	1,257 (29.1)
Region				
Central	409 (20.3)	375 (18.6)	2,007 (46.5)	1,625 (37.7)
East	1,311 (65.1)	1,175 (58.4)	1,350 (31.3)	1,058 (24.5)
West	293 (14.6)	262 (13.0)	959 (22.2)	860 (19.9)
Marriage				
Married	1,456 (72.3)	1,328 (66.0)	3,495 (81.0)	2,871 (66.5)
Others (single, divorced or widowed)	557 (27.7)	484 (24.0)	821 (19.0)	672 (15.6)
Education level				
Middle school and below	111 (5.5)	92 (4.6)	2,152 (49.9)	1,715 (39.7)
High school	585 (29.1)	517 (25.7)	835 (19.3)	686 (15.9)
Bachelor	1,214 (60.3)	1,107 (55.0)	1,281 (29.7)	1,100 (25.5)
Master and above	103 (5.1)	96 (4.8)	48 (1.1)	42 (1.0)
Employment				
Employed	1,714 (85.1)	1,561 (77.6)	2,249 (52.1)	1,920 (44.5)
Others	299 (14.9)	251 (12.5)	2,067 (47.9)	1,623 (37.6)
Annual household income (CNY)				
≤ 50,000	207 (10.3)	173 (8.6)	1,537 (35.6)	1,245 (28.9)
50,000–100,000	490 (24.3)	435 (21.6)	1,420 (33.0)	1,194 (27.7)
100,000–150,000	489 (24.3)	435 (21.6)	709 (16.4)	591 (13.7)
150,000–200,000	395 (19.6)	367 (18.2)	268 (6.2)	200 (4.6)
200,000–300,000	284 (14.1)	268 (13.3)	251 (5.8)	205 (4.8)
≥300,000	148 (7.4)	135 (6.71)	131 (3.0)	108 (2.5)
Household size				
1–2	209 (10.4)	168 (8.0)	1,372 (31.8)	1,069 (24.8)
3–4	1,225 (60.9)	1,113 (52.9)	1,711 (39.6)	1,427 (33.1)
≥5	579 (28.8)	531 (25.3)	1,233 (28.6)	1,047 (24.3)
Has the elderly at home				
Yes	906 (45.0)	832 (41.3)	2,514 (58.2)	2,064 (47.8)
No	1,107 (55.0)	980 (48.7)	1,801 (41.8)	1,479 (34.3)
Health status				
Very good/good	1,366 (68.0)	1,262 (62.7)	4,095 (95.0)	3,371 (78.1)
Normal/poor	647 (32.0)	550 (27.3)	221 (5.0)	172 (4.0)
Has chronic disease				
Yes	254 (12.6)	237 (11.8)	1,396 (32.3)	1,117 (25.9)
No	1,759 (87.4)	1,575 (78.2)	2,920 (67.7)	2,426 (56.2)

In the on-site survey, 4,316 respondents were located in five provincial administrative regions in China, and around half of them (43.4%) were above 60 years old, showing a sample with more senior citizens. Among all respondents, 52.1% were employed, 46.5% lived in central China, and 64.2% lived in urban areas. The majority (69.2%) of respondents had a high school and below degree. Most of them (95.0%) thought their health status was good or very good, and 67.7% did not report any chronic diseases. Most of the respondents (68.5%) had an annual family income of less than CNY 100,000 (USD 15,340). Around one-third of the respondents lived in a family with 1–2 members (31.8%), and 58.2% of the respondents lived with the elderly.

### Comparison of Acceptance of COVID-19 Vaccination Between the Two Surveys

Among the 2,013 respondents in the online survey, the proportion of general respondents who accepted COVID-19 vaccination was 90.0%, higher than that of the on-site survey (82.1%). Further comparing demographic characteristics of those who accepted the vaccination, differences were observed as 88.5% of online respondents who accepted to be vaccinated were below 60 years old, but the proportion was 48.2% in the on-site survey. Total 58.4% of online respondents who accepted were located in eastern China, while there were 24.5% in eastern China and 37.7% in central China in the on-site survey. Total 77.6% of online respondents who accepted were employed, but in the on-site survey, 44.5% respondents were employed. The results showed the differences in vaccination acceptance across differentiated demographic locations and socioeconomic characteristics.

### Influencing Factors of Vaccine Acceptance Between the Two Surveys

Since the on-site results showed a lower level of acceptance of vaccination, the multivariate logistic regression was then performed between the online survey group and the on-site survey group to identify influencing factors of vaccination acceptance. Data from the online and on-site samples were pooled together in the logistic regression. We also conducted logistic regressions for the online and on-site samples, respectively. The results of regression models are presented in Table 2. The regression of pooled data showed that compared with online respondents, those in the on-site survey had significantly lower vaccine acceptance (OR: 0.49, 95% CI: 0.39–0.63). In addition, those aged above 60 years (OR: 0.73, 95% CI: 0.54–0.98) and considered their health status as normal or poor (OR: 0.58, 95% CI: 0.44–0.75) were less intended to accept vaccination. In contrast, those located in eastern (OR: 1.27, 95% CI: 1.07–1.52) or western China (OR: 2.10, 95% CI: 1.68–2.62), having a bachelor's degree (OR: 1.35, 95% CI: 1.07–1.70) or master's degree or above (OR: 1.78, 95% CI: 0.95, 3.31), having the elderly at home (OR: 1.37, 95% CI: 1.16–1.62), and being unemployed (OR: 1.45, 95% CI: 1.22–1.73) were more likely to accept vaccination. In the logistic regression for the online and on-site sample alone, the household income became a significant influencing factor of vaccination acceptance. In the online survey regression, higher household income ranging from CNY 150,000 to 200,000 (OR: 1.806, 95% CI: 0.971–3.359) and from CNY 200,000 to 300,000 (OR: 2.099, 95% CI: 1.025–4.298) led to stronger intention to be vaccinated. However, in the on-site survey regression results, those with household income from CNY 150,000 to 200,000 (OR: 0.562, 95% CI: 0.398, 0.794) were less intended to be vaccinated.

**Table 2:** Influencing factors of vaccine acceptance in the pooled, online, and on-site samples.

**Survey type**	**Online vs. on-site**		**Online**		**On-site**	
**Characteristics**	**OR**	**95%CI**	**OR**	**95%CI**	**OR**	**95%CI**
Survey type						
Online	Ref					
On-site	0.494^*^	(0.387, 0.630)^*^				
Age group						
18~30	Ref		Ref		Ref	
31~40	0.953	(0.740, 1.226)	0.931	(0.607, 1.426)	0.862	(0.621, 1.197)
41~50	0.838	(0.638, 1.102)	0.864	(0.528, 1.414)	0.776	(0.550, 1.096)
51~60	0.952	(0.704, 1.288)	0.553^*^	(0.302, 1.012)^*^	1.000	(0.696, 1.437)
>60	0.727^*^	(0.536, 0.984)^*^	1.077	(0.306, 3.786)	0.662^*^	(0.464, 1.944)^*^
Gender						
Female	Ref		Ref		Ref	
Male	1.030	(0.893, 1.188)	1.043	(0.766, 1.421)	1.013	(0.86, 1.192)
Region						
Central	Ref				Ref	
East	1.274^*^	(1.070, 1.518)^*^	1.431^*^	(0.944, 2.171)	1.21^*^	(0.99, 1.478)^*^
West	2.098^*^	(1.677, 2.624)^*^	1.161	(0.753, 1.792)	2.348^*^	(1.809, 3.046)^*^
Living residence						
Urban	Ref		Ref		Ref	
Rural	0.982	(0.834, 1.156)	0.771	(0.495, 1.201)	1.007	(0.842, 1.203)
Marriage						
Married	Ref		Ref			
Others (single, divorced or widowed)	1.101	(0.914, 1.327)	1.248	(0.824, 1.889)	0.982	(0.791, 1.219)
Education						
Middle school and below	Ref		Ref		Ref	
High school	1.069	(0.868, 1.316)	1.31	(0.712, 2.413)	1.083	(0.863, 1.360)
Bachelor	1.348^*^	(1.070, 1.697)^*^	1.616	(0.872, 2.996)	1.308^*^	(1.005, 1.703)^*^
Master and above	1.775^*^	(0.950, 3.316)^*^	1.903	(0.690, 5.247)	1.628	(0.657, 4.035)
Employment						
Employed	Ref		Ref		Ref	
Others	1.451^*^	(1.216, 1.730)^*^	1.551^*^	(1.016, 2.369)^*^	1.363^*^	(1.120, 1.658)^*^
Household income						
≤ 50,000	Ref		Ref		Ref	
50,000–100,000	1.028	(0.852, 1.240)	1.344	(0.813, 2.220)	1.028	(0.838, 1.260)
100,000–150,000	0.935	(0.744, 1.175)	1.144	(0.673, 1.943)	0.959	(0.737, 1.247)
150,000–200,000	0.822	(0.615, 1.099)	1.806^*^	(0.971, 3.359)^*^	0.562^*^	(0.398, 0.794)^*^
200,000–300,000	1.085	(0.778, 1.514)	2.099^*^	(1.025, 4.298)^*^	0.863	(0.585, 1.273)
≥300,000	0.940	(0.618, 1.431)	1.298	(0.595, 2.832)	0.918	(0.544, 1.550)
Household size						
1–2	Ref		Ref		Ref	
3–4	1.279^*^	(1.063, 1.539)^*^	1.806^*^	(1.166, 2.795)^*^	1.185	(0.965, 1.455)
≥5	1.385^*^	(1.122, 1.709)^*^	1.691^*^	(0.974, 2.936)^*^	1.395^*^	(1.106, 1.760)^*^
Has the elderly at home						
Yes	Ref		Ref		Ref	
No	1.368^*^	(1.156, 1.618)^*^	1.388^*^	(0.964, 1.999)^*^	1.373^*^	(1.129, 1.669)^*^
Health status						
Very good/good	Ref		Ref		Ref	
Normal/poor	0.578^*^	(0.445, 0.751)^*^	0.497^*^	(0.311, 0.792)^*^	0.741	(0.204, 2.701)
Has chronic disease						
Yes	Ref		Ref		Ref	
No	1.274^*^	(1.061, 1.529)^*^	2.415^*^	(1.358, 4.295)^*^	1.157	(0.949, 1.410)

* *P < 0.05*.

### The Adjusted Acceptance and Influencing Factors Under PSM

Table 3 presents the adjusted results generated from the PSM using the nearest neighbor matching method among respondents in the online and on-site surveys. Before matching these two samples, the unmatched vaccine acceptance was 90.0% for the online survey and 82.1% for the on-site survey, showing a difference of 7.9%. In the PSM analysis, the individuals in the control group (the on-site sample) with the smallest difference in propensity score from those in the treatment group (the online sample) were compared. Controlled individuals were identified according to the information of treated individuals, and all treated individuals were paired successfully, so their information could be fully used.

**Table 3:** The results of propensity score matching using a nearest neighbor matching method.

**Variable sample**		**Online survey (*n* = 2,013)**	**On-site survey (*n* = 4,316)**	**ATT**	**Standard error**	***t*-value**
Acceptance to vaccination	Unmatched	0.900	0.821	0.079	0.010	8.170^*^
	Under nearest neighbor matching	0.900	0.786	0.114	0.114	3.240^*^

* *P < 0.05*.

**Figure 1:**
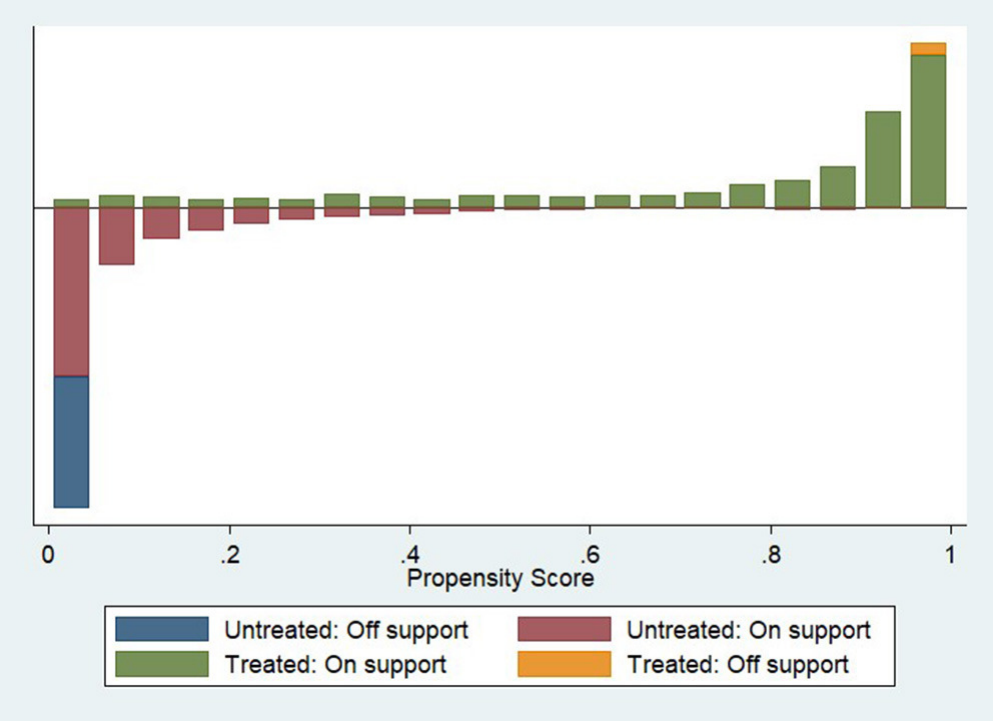
The common range of propensity scores.

**Figure 2:**
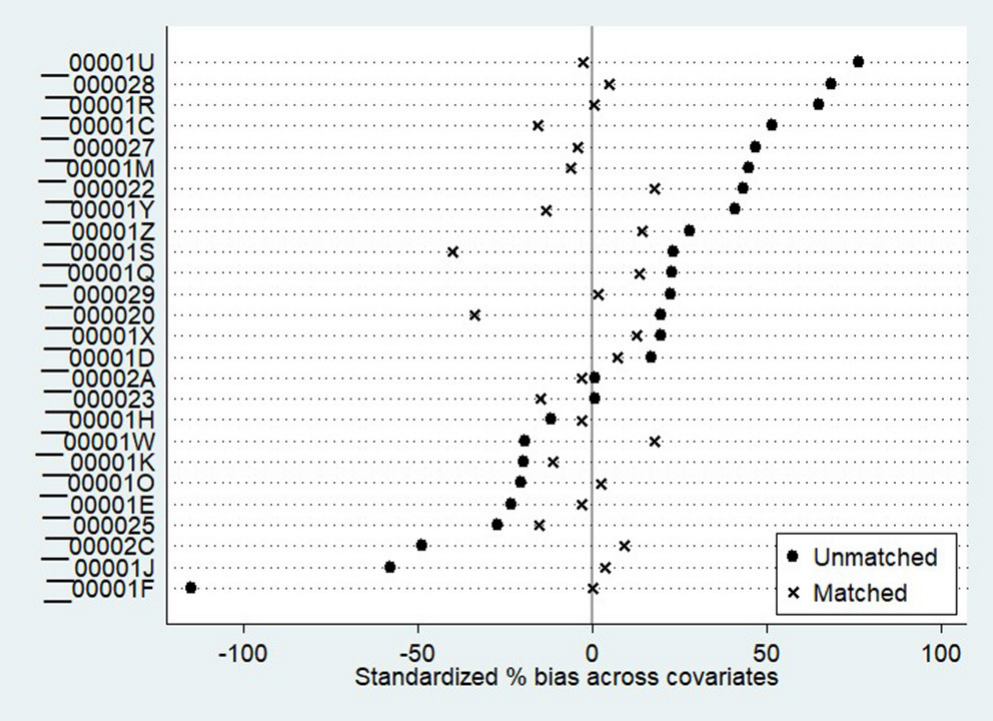
The standardized bias across covariates.

After conducting PSM, the vaccination acceptance of the on-site survey was declined to 78.6%, with the difference increasing to 11.4% compared with the online survey. The average treatment effect for the treated (ATT) value was significant at a 5% significance level, indicating a difference in vaccination acceptance between the two surveys. Table 4 and Figure 1 show the common range of observed values under PSM. Of the 6,307 observations, 1,182 were not within the common range (off support), and the remaining 5,125 were within the common range (on support). Table 5 demonstrates the Propensity Score Testing (PSTEST) results, which examined whether the matched results could balance the differences of the values and checked whether there was a significant difference in the matched covariates between the two survey groups. For respondents who aged from 31 to 60 years, obtained high school or master and above degrees, being with annual household income more than CNY 50,000, with household size more than 2, not living with the elderly at home, with normal or poor health status, and without any chronic diseases, significant differences existed among these factors between the online and on-site survey groups. Figure 2 denotes the standardized bias across the covariates according to Table 4.

**Table 4:** The common support for domain hypothesis testing under propensity score matching.

	**Off support**	**On support**	**Total**
On-site	1,131	3,163	4,294
Online	51	1,962	2,013
Total	1,182	5,125	6,307

**Table 5:** PSTEST results to examine whether the matched results could balance the differences of covariates under online and on-site surveys.

	**Mean**			***T*** **-test**	
**Variable**	**Online survey (*n* = 2,013)**	**On-site survey (*n* = 4,316)**	**%bias**	***t*-value**	***P*-value**
Age group					
18~30	Ref				
31~40	0.358	0.380	−5.300	−1.440	0.151
41~50	0.183	0.161	6.300	1.840	0.065
51~60	0.069	0.089	**−6.800**	−2.390	0.017^*^
> 60	0.018	0.021	−1.000	−0.810	0.417
Gender					
Female	Ref				
Male	0.518	0.510	1.600	0.500	0.620
Region					
Central	Ref				
East	0.208	0.218	−2.200	−0.750	0.455
West	0.149	0.157	−2.200	−0.750	0.453
Living residence					
Urban	Ref				
Rural	0.833	0.845	−2.700	−0.970	0.330
Marriage					
Married	Ref				
Others (single, divorced or widowed)	0.729	0.720	2.200	0.650	0.519
Education					
Middle school and below	Ref				
High school	0.284	0.242	**10.000**	3.040	0.002^*^
Bachelor	0.610	0.623	−2.700	−0.830	0.404
Master and above	0.049	0.088	**−22.500**	−4.830	<0.001^*^
Employment					
Employed	Ref				
Others	0.848	0.859	−2.600	−1.000	0.318
Household income					
≤ 50,000	Ref				
50,000–100,000	0.249	0.185	**14.100**	4.820	<0.001^*^
100,000–150,000	0.240	0.197	**10.600**	3.240	<0.001^*^
150,000–200,000	0.194	0.238	**−13.500**	−3.380	<0.001^*^
200,000–300,000	0.139	0.087	**17.500**	5.150	<0.001^*^
≥300,000	0.073	0.113	**–**17**.700**	−4.230	<0.001^*^
Household size					
1–2	Ref				
3–4	0.609	0.518	**18.600**	5.760	<0.001^*^
≥5	0.286	0.352	**−14.700**	−4.480	<0.001^*^
Has the elderly at home					
Yes	Ref				
No	0.445	0.516	**−14.400**	−4.490	<0.001^*^
Health status					
Very good/good	Ref				
Normal/poor	0.268	0.196	**20.600**	5.380	<0.001^*^
Has chronic disease					
Yes	Ref				
No	0.129	0.107	**5.400**	2.140	0.032^*^

*The bold values means that the standardized mean bias percentage of that variable is larger than 5%*.

* *P < 0.05*.

## Discussion

To examine the public acceptance of COVID-19 vaccination in China and its differences under two survey methodologies, online and on-site surveys were conducted simultaneously during the well-contained phase of the pandemic (before the approval of COVID-19 vaccines). The present study revealed that the acceptance rate of COVID-19 vaccination among Chinese adults was as high as 90.0% in the online survey, demonstrating a minor reduction compared with the rate in the severe epidemic phase (91.9% in March 2020) (9). Meanwhile, the acceptance rate of vaccination was lower at 82.1% in the on-site survey. The difference suggested that even in the same phase of the pandemic using the same questionnaire questions, different survey methods could generate varied acceptance rates of vaccination. To interpret the difference in vaccination acceptance between the two surveys, several influencing factors should be considered, such as the demographic characteristics and self-perception of health status. According to the PSM results, the matched results indicated that the on-site acceptance rate declined to 78.6%, with an additional 3.5% in the acceptance gap compared to the online survey. A systematic difference in major outcomes and influencing factors indicated that different types of surveys could address discrepant results from the differentiated sampling and investigation measures.

Since survey techniques are widely used to explore human behaviors (35), they are frequently adopted in social and psychological research studies. By comparison, online surveys could be rapidly deployed and completed by respondents, particularly when disseminated via social media, web-based platforms, or where an incentive is offered for completion (36). Minimum cost would be incurred in online surveys because the questionnaire delivery and response can be completed automatically, reducing the payments to face-to-face investigators. Besides, the online survey could make it easier to understand complex or lengthy questions, which also avoid the complex process of coding and data cleaning to decrease data entry errors (25, 36, 37). However, the lack of an on-site interviewer can be a disadvantage in closed-ended online surveys, as respondents could not be clarified of unfamiliar or ambiguous terms in questionnaires (36). Moreover, the accumulation of biased or non-representative responses is also a drawback of the online surveys as those who lacked Internet access would not be captured, such as the elderly, those with lower income, or reside in remote rural areas (37, 38). For example, in the present study, the vaccination acceptance was higher among those aged below 60 years old in the online survey, compared with the on-site survey. After applying the PSM analysis, we deduced that the lack of access to web-based platforms might result in a lower level of participation of the elderly, further causing the bias of higher vaccination acceptance in the online survey.

During the pandemic in most countries, although the online survey is one of the few choices to conduct public investigations given the limitation of social distancing, self-administered online questionnaires are actually not a very useful tool for approaching illiterate or non-literate populations or those who cannot proficiently use technologies. In contrast, on-site surveys could effectively assign the sample by different regions and populations based on the socioeconomic development and specific research purposes, supporting to collect more representative responses. The potential sample bias could also be addressed by conducting on-site surveys, which could decline the possibility of over-representation to a particular viewpoint and the survey fraud (e.g., duplicate responses, false information, or deliberately exaggerated responses) (37, 39, 40). In the present study, PSM analysis results showed that the on-site vaccination acceptance rate was dropped to 78.6% after matching, with significant biases in factors, such as age, household income, household size, and health status. It indicates that systematic differences caused by the online investigation approach actually existed in evaluating public acceptance of COVID-19 vaccination, and its influencing factors were converged to a higher level of acceptance. The virtual-high results might further mislead the authority that most Chinese citizens were highly willing to get vaccinated, which is contrary to the fact that the Chinese government still needs to raise public awareness and acceptance toward COVID-19 vaccination.

Currently, it is not convenient to conduct on-site surveys in other countries, so most research groups implemented online surveys rather than on-site ones to collect public acceptance of COVID-19 vaccination. Since we conducted the surveys during the well-contained phase of the pandemic in China where the pandemic was effectively controlled, we were able to implement an on-site survey across five provinces/municipalities and compare the online and on-site results in terms of vaccine acceptance. Although PSM analysis may not completely solve the endogenous problem, it can effectively alleviate the deviation caused by self-selection. It can be illustrated from the survey and PSM results that the systematic difference of vaccine acceptance existed in the two types of surveys, but the difference gap was relatively small (90.0% compared with 78.6%).

Compared with other countries, even in the well-contained phase, the acceptance of vaccination in China remained higher. Studies reported that public acceptance of COVID-19 vaccination ranged from 62 to 80% in some European countries, among which Denmark and the UK had the highest acceptance (80%), while France (58.9–62%) and Italy (59%) had the lowest. While in Asian countries, the acceptance rates were relatively higher, as 79.8% in South Korea, 67–93.3% in Indonesia, and 94.3% in Malaysia (10–13, 41–45). Based on our findings, since an online survey might address the relatively higher acceptance rate of COVID-19 vaccination, it is possible that the real public acceptance of COVID-19 vaccination in those countries is lower than the reported survey results. Therefore, specific population groups are suggested to be considered in the online surveys to improve the representativeness of the study sample, by either combing with on-site interviews to reach specific population groups, or adjusting the outcomes through statistical approaches, such as PSM (37). Additionally, online questionnaires are recommended to improve the explicit delivery with some interpretations for key questions (36, 37, 40) and set duplicated questions to check for internal consistency. Encouraging the participants by giving specific incentives could be effective to improve the quality of online surveys, especially when the targeted participants have no or low level of income.

For limitations of this study, firstly, our study did not stratify the sample size by urban and rural areas of western China in the on-site survey, while geographic locations will inevitably affect the vaccination acceptance. It is due to that in western China, we conducted the on-site survey in Chongqing, most part of which is urban area with higher vaccination acceptance compared with rural areas. However, in central and eastern China, we conducted the survey in both urban and rural areas. This could explain why the acceptance rate in the western area was higher in the present study. Secondly, we did not conduct on-site surveys in all 31 provincial administrative regions as done in an online survey, but we collected samples from eastern, central, and western China in both the surveys, which help to minimize the deviations caused by geographical location differences. Thirdly, there was a gap in a sample size of the online (*n* = 2,013) and on-site survey (*n* = 4,316), but we have tried our best to collect data during the exact same time period for the two surveys, with sample size for each was over 2,000. This was due to some objective restrictions in the web-based survey method. According to Couper, web-based surveys present challenges particularly in sampling, coverage, non-response, and measurement errors (46). Without offering a paper-based questionnaire, a small but potentially important group of populations who did not have access to the Internet or did not receive the notification of online questionnaires would likely be missing with potentially biased estimates in the results (47). Fourthly, based on the fact that there were more senior participants in the on-site survey compared with the online survey, the selection bias within the results would possibly mislead the analysis for public acceptance of vaccination. To minimize the bias, we adjusted the results by applying the PSM methodology to estimate treatment effects when the systematic differences between the two surveys were not random, and all treated individuals were paired successfully.

## Conclusion

This study simultaneously conducted the online and on-site survey toward the acceptance of COVID-19 vaccination among the Chinese population during the well-contained phase of the pandemic, which was the first large-scale on-site survey of COVID-19 vaccination acceptance and the first study to compare the simultaneous online and on-site results, both in China and in the world. From our study, a 7.9% gap was reported between the results of online and on-site surveys in a public acceptance rate of COVID-19 vaccination, and the gap became larger to 11.4% after PSM adjusting. Besides, multivariate logistic regression was conducted with the variables of age-distribution, region, education, employment status, household income, household size, and health status, which would affect vaccination acceptance. During the pandemic, it is not convenient to conduct on-site surveys in other countries, so most research groups implemented online surveys rather than on-site ones. Therefore, based on the findings of our study, statistical approaches could help to reduce the biases and enhance the rigor of online survey results to make them closer to the results generated from the on-site field survey. However, although statistical approaches could be applied to adjust the online survey results, we still recommend global researchers to combine online surveys with some small-scaled on-site surveys to ensure the capture of valid responsiveness and appropriate sample stratification.

## Data Availability Statement

The raw data supporting the conclusions of this article will be made available by the authors, without undue reservation.

## Ethics Statement

The study was approved by Peking University Institutional Review Board (IRB00001052-20011). All participants gave informed consent before taking part.

## Author Contributions

LL and HFa conceived the presented idea. LL, HFa, JW, XL, and HZ developed the design and the questionnaire. LL, LC, and HL conducted the on-site survey. HFa, YL, and XL performed the analysis. YL and XL wrote the first draft. XM, HFa, LL, LC, HL, HFe, RJ, and JG commented on the first draft and revised the manuscript. All authors discussed the results and contributed to the final manuscript.

## Conflict of Interest

The authors declare that the research was conducted in the absence of any commercial or financial relationships that could be construed as a potential conflict of interest.

## Publisher's Note

All claims expressed in this article are solely those of the authors and do not necessarily represent those of their affiliated organizations, or those of the publisher, the editors and the reviewers. Any product that may be evaluated in this article, or claim that may be made by its manufacturer, is not guaranteed or endorsed by the publisher.
